# Ventriculoatrial shunt remains a safe surgical alternative for hydrocephalus: a systematic review and meta-analysis

**DOI:** 10.1038/s41598-024-62366-8

**Published:** 2024-08-09

**Authors:** Enrico Lo Bue, Alberto Morello, Jacopo Bellomo, Leonardo Bradaschia, Filippo Lacatena, Stefano Colonna, Alessandro Fiumefreddo, Lennart Stieglitz, Luca Regli, Michele Maria Lanotte, Diego Garbossa, Fabio Cofano

**Affiliations:** 1 Neurosurgery Unit, Department of Neuroscience “Rita Levi Montalcini”, AOU Città della Salute e della Scienza di Torino, University Hospital, University of Turin, 10124 Turin, Italy; 2 Department of Neurosurgery, University Hospital Zurich, Zurich, Switzerland; 3 Clinical Neuroscience Center, University of Zurich and Swiss Federal Institute of Technology Zurich, Zurich, Switzerland; 4 Stereotactic and Functional Neurosurgery Unit, Department of Neuroscience “Rita Levi Montalcini”, AOU Città della Salute e della Scienza di Torino, University Hospital, University of Turin, Turin, Italy

**Keywords:** Ventriculoatrial, Ventriculo-atrial, Ventriculo-peritoneal, Shunt, Hydrocephalus, Neuroscience, Diseases of the nervous system

## Abstract

Hydrocephalus is a commonly encountered pathology in the neurosurgical practice. Since the first permanent ventriculo-subarachnoid-subgaleal shunt described by Mikulicz in 1893, there were multiple attempts to find solutions for draining the excess production/less reabsorption of the cerebrospinal fluid (CSF) from the brain. Nowadays, the most common technique is the ventriculoperitoneal shunt (VPS), whereas the ventriculoatrial shunt (VAS) is applied only in some rare conditions. To date there are still no specific guidelines or strong evidence in literature that guide the surgeon in the choice between the two methods, and the decision usually relies on the confidence and expertise of the surgeon. Considering the lack of established recommendations, this systematic review and meta-analysis aims to evaluate the effectiveness and safety of these two shunting techniques. This systematic review was conducted following the PRISMA protocol (Preferred Reporting Items for Systematic Reviews and Meta-Analyses). No chronological limits of study publications were included. Prospective and retrospective clinical studies, and reports of case series with at least five patients per group and reporting data on comparison between VAS and VPS techniques were eligible for inclusion. Nine studies reporting 3197 patients meeting the inclusion and exclusion criteria were identified and included in the quantitative synthesis. The risk of shunt dysfunction/obstruction was significantly lower in the VAS group [odds ratio (OR) 0.49, 95%-CI 0.34–0.70, I^2^ 0%]. The risk of infection was not significantly different between the two groups (OR 1.02, 95%-CI 0.59–1.74, I^2^ 0%). The risk of revision was not significantly different between the two groups; however, the heterogeneity between the studies was significant (OR 0.73, 95%-CI 0.36–1.49, I^2^ 91%). Additionally, the risk of death was not significantly different between the two groups; however, the heterogeneity between the studies was high (OR 1.93, 95%-CI 0.81–4.62, I^2^ 64%). VAS remains a safe surgical alternative for hydrocephalus. The results of this study highlight a lower risk of shunt dysfunction/obstruction variable in the VAS group, with no significant statistical differences regarding the occurrence of at least one infection-related complication. In consequence, the choice between these two techniques must be tailored to the specific characteristics of the patient.

*Protocol Registration*: The review protocol was registered and published in Prospective Register of Systematic Reviews (PROSPERO) (www.crd.york.ac.uk/PROSPERO) website with registration number: CRD42023479365.

## Introduction

In the 1950s the creation of a valve system with the ability to regulate opening pressure and prevent CSF reflux in the brain initiated a new era of surgical treatments for hydrocephalus. Consequently, it led to the development of the ventriculoatrial shunt (VAS) in the 1960s^1^ and the ventriculoperitoneal shunt (VPS) in the 1970s^2^. Originally, VAS was considered superior to VPS because of the polyethylene tube of the latter, which had unacceptable rates of peritonitis and distal failure. However, it was quickly noted that VAS carried significant concerns regarding the recognition of various serious and even fatal complications, such as atrial thrombi, pulmonary embolism, bacteremia, pulmonary hypertension, and cor pulmonale^3^.

Through the years, VPS has steadily gained ground compared to VAS due to a multitude of factors, including the simplicity of the surgical technique and a faster learning curve. In addition, the high peritoneal absorptive capacity, as demonstrated by its use in peritoneal dialysis, allows the placement in pediatric population of additional length of catheter for growth avoiding lengthening procedures^4^. Additionally, VAS represents a last resort treatment for hydrocephalus, notably when VPS is not feasible. Furthermore, VAS may be underutilized due to the technical preferences of neurosurgeon, and less tendency of young neurosurgeons to learn and master the technical procedure^5^.

To the best of the author’s knowledge, there are no clear guidelines clarifying the use of VAS or VPS as first surgical solution for shunt placement, demanding the choice to the surgeon or to internal guidelines of each institution. Since the lack of established recommendation, this systematic review and meta-analysis aims to evaluate the effectiveness and safety of these two shunting techniques.

## Materials and methods

### Literature search

This systematic review was conducted following the PRISMA protocol (Preferred Reporting Items for Systematic Reviews and Meta-Analyses)^6^. Potentially relevant literature was retrieved from PubMed/MEDLINE, Embase, and the Cochrane Library. The final search was conducted on the 20th of September 2023. Detailed search strategy is reported in Supplementary Material 1. Word variations and exploded medical subject headings were searched for whenever feasible.

### Inclusion and exclusion criteria

Comparative studies in English language that met the following PICO (Patient, Intervention, Comparison, Outcome) criteria were considered eligible. Patients: individuals with symptomatic hydrocephalus. Intervention: VAS. Comparison: VPS. Outcomes: surgical revision, shunt dysfunction, infection, mortality.

No chronological limits of study publications were adopted. Prospective and retrospective clinical studies, and reports of case series with at least five patients per group and reporting data on comparison between VAS and VPS techniques and reporting at least one outcome of interest were eligible for inclusion. Meta-analyses, case reports, or studies with less than 5 patients per group, cadaver studies, laboratory and animal studies were excluded. Studies including only one surgical method or other possible shunting techniques such as ventriculo-ureteral, ventriculo-gallbladder, ventriculo-pleural cavity or ventriculo-subgaleal shunt, were not included.

### Screening and full-text review

Title and abstract screening, full-text review, and data extraction were undertaken in parallel by two reviewers (F.L. and L.B.). Disagreements at any stage were resolved by discussion and consensus. The main disagreements concerned the absence of clear comparative studies between the VAS and VPS techniques; in this case they were resolved by the involvement of a third reviewer (A.M.). The process was carried out using Rayyan^7^.

### Data extraction

Several items were considered in the evaluation of VAS/VPS surgical techniques and were divided in two main categories: patient demographics and surgery characteristics. In the first group sex, mean age at first placement of the CSF shunt system, and the etiology of the hydrocephalus were investigated. In the second group data on primary surgical choice, VAS/VPS short- and long-term complications, resolution of the hydrocephalus, number of revisions, and the mean time at first revision of the CSF diversion system were collected. The following data were extracted: author name, publication year, the country of studies, study design, sample size, age, etiology of hydrocephalus, size of surgical groups (VAS/VPS), median follow-up time, and outcome measures that were reported as frequencies during follow-up time.

### Risk-of-bias assessment

The Cochrane risk-of-bias tool for nonrandomized studies of interventions (ROBINS-I tool) was used for risk-of-bias assessment of the included studies^8^. This was performed by two authors (J. B., and A. M.).

### Statistical analysis

The statistical analysis was carried out with the statistical program R studio (Posit Software, PBC formerly R Studio, version 02.07.2022). The baseline characteristics of the included studies were analyzed using descriptive statistics. The meta-analysis was performed using the package meta (version 6.5-0, published 2023-06-07). A random effect model for the meta-analysis was conducted because of the methodological and clinical differences among the studies. The odds ratio (OR) of frequency data with the corresponding 95% CI were pooled by the inverse-variance test. The I2 test was used to capture the between studies’ heterogeneities, which refers to the proportion of total variation because of the differences among included studies instead of sampling error. All statistical tests were two-tailed, and the significance level was set at *P* value < 0.05.

## Results

### Literature search

**Figure 1:**
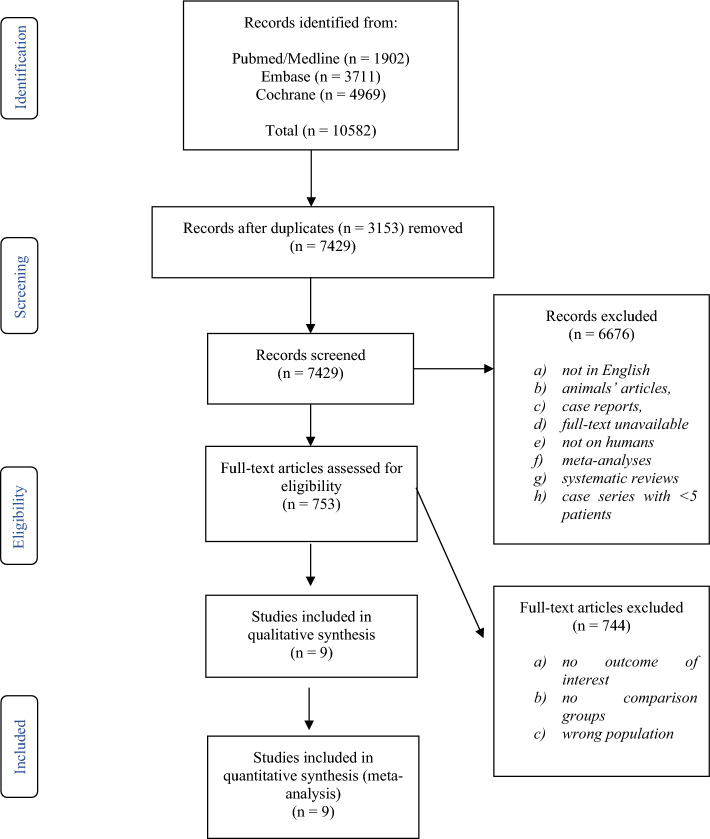
PRISMA flowchart.

As illustrated in the PRISMA flowchart of Fig. 1**,** the PubMed/MEDLINE, Embase and Cochrane Library search provided 10,582 articles. After duplicate removal (n = 3153), 7429 records were screened, and 753 were then assessed for eligibility through full-text screening. Finally, 9 studies meeting the inclusion and exclusion criteria and reporting on 3197 patients were identified and included in the quantitative synthesis. Sufficient data was available to perform meta-analysis for surgical revision, shunt dysfunction/obstruction, infection, and mortality.

### Study characteristics and quality

Table 1 lists the main characteristics of the included studies, including publication year, study design and sample size, etiology of hydrocephalus, size of surgical groups, median follow-up time (in months), mean age at surgery (in years), and the risk of bias evaluated with ROBINS-I tool. These 9 studies yielded 3197 patients with hydrocephalus, of whom 1338 (42%) and 1798 (56%) received VAS and VPS surgery, respectively. Three studies (Olsen and Frykberg, Fernell et al., Keucher and Maeley) investigated pediatric patient cohorts, and three studies (Lam and Villemure, McGovern et al., and Hung et al.) adult patient cohorts. The remaining three studies (Ignelzi and Kirsch, Borgbjerg et al., and Rymarczuk et al.) included a mixed cohort of pediatric and adult patients. Apart from the study of Hung et al., comparing VPS and VAS in a cohort of idiopathic normal pressure hydrocephalus (NPH), all the other studies included patients with different causes of hydrocephalus. All the included studies had a retrospective study design, and they were scored with an overall serious to critical risk of bias according to the Cochrane’s ROBINS-I tool (Table 1). In Table 2 the frequencies of the investigated outcome variables (revision surgery, shunt dysfunction/obstruction, infection-related complication, death) in the included studies are summarized. In this context, it is worth to mention that the studies of Ignelzi and Kirsch, Fernell et al., Lam and Villemure, and Borgbjerg et al. did not report the median follow-up time between the two surgical groups. Additionally, the median follow-up time showed a consistent difference in the studies of Keucher and Maeley, shorter in the VPS group, and Hung et al., shorter in the VAS group. In the studies of Fernell et al. and Keucher and Maeley the total amount of surgical revision was reported, thus not allowing to differentiate the number of patients that needed at least one surgical revision. Over the years, a trend towards performing VPS as primary surgical treatment for hydrocephalus was observed. Indeed, in three most recent studies (McGovern et al., Hung et al. and Rymarczuk et al.) VAS was considered and performed as primary shunt treatment option only in case of contraindications for VPS, such as previous abdominal surgery, suspected increased intra-abdominal pressure, history of peritonitis, abdominal trauma, or other abdominal infections that could threaten the shunt sterility or challenge its positioning due to tissue adherence. Conversely, in older studies the decision to perform VPS or VAS relied more on the surgeon’s preference and experience rather than on patient-related factors.

**Table 1:** Principle characteristics of the included studies.

Authors	Publication date	Study design	Nr. of included patients	Hydrocephalus etiology	Shunt treatment		Median Follow-up time (in months)		Mean age at surgery (in years)		Overall risk of bias
					VPS	VAS	VPS	VAS	VPS	VAS	
Ignelzi and Kirsch	1975	Retrospective study	300	Aqueductal stenosis (54), aqueductal stenosis and myelomeningocele (43), neoplasm (55), communicating hydrocephalus not further specified (148)	114	177	NA	NA	NA%	NA%	Critical
Olsen and Frykberg	1983	Retrospective study	172	Spina bifida (55), CNS malformation (46), infection (12), perinatal factors (33), postnatal factors (2), neoplasm (2), unknown (22)	69	103	57	46	0.38	0.41	Serious
Fernell et al	1985	Retrospective study	259	Aqueductal stenosis (84), perinatal complications (69), CNS anomalies (49), congenital or neonatal infection (30), unknown (27)	133	80	NA	NA	0.60	0.90	Critical
Keucher and Maeley	1979	Retrospective study	228	Myelomeningocele (128), aqueductal stenosis (39), communicating hydrocephalus not further specified (31), infection (12), Dandy-Walker syndrome (9), unknown (9)	81	147	60	97	0.22	0.26	Serious
Lam and Villemure	1997	Retrospective study	128	Normal pressure hydrocephalus (55), neoplasm (37), haemorrhage (17), aqueductal stenosis (4), trauma (4), infection (3), congenital (2), unknown (6)	73	49	NA	NA	NAΔ	NAΔ	Critical
Borgbjerg et al	1998	Retrospective study	883	NA	366	517	NA	NA	27.00	27.00	Serious
McGovern et al	2014	Retrospective study	187	Idiopathic normal pressure hydrocephalus (168), neoplasm (13), aqueductal stenosis (2), CNS cyst (2), trauma (1), Chiari spectrum (1)	157	30	34	42	76.00	73.70	Serious
Hung et al	2017	Retrospective study	496	Idiopathic normal pressure hydrocephalus (496)	346	150	41	15	73.00	74.00	Serious
Rymarczuk et al	2019	Retrospective study	544	Haemorrhage (128), neoplasm (79), spina bifida (72), congenital (45), infection (20), Dandy-Walker syndrome (16), aqueductal stenosis (15), trauma (15), Chiari spectrum (13), encephalocele (7), pseudotumor (6), schizencephaly (6), arachnoid cyst (6), vascular lesion (7), craniofacial syndrome (4), Aicardi syndrome (1), errors of metabolism (2), fibrous dysplasia (1), unknown (21)**§**	459	85**§**	71	64	2.30	7.80	Serious

The risk of bias was assessed using the ROBINS-I tool. %, the patients included in the study by Ignalzi and Kirsch represent a mixed pediatric and adult cohort with the age ranging from 1 day to 90 years; no data about the mean age value is provided. Δ, the patients included in the study by Lam and Villemure represent an adult cohort; no data about the mean age value is provided. §, 80 of the 85 patients received VAS as secondary treatment option after VPS failure. CNS indicates central nervous system; VPS, ventriculoperitoneal shunt; VAS, ventriculoatrial shunt.

**Table 2:** Occurrence of the selected outcomes of interest in the included studies.

Authors	Nr. of patients with at least one revision surgery		Nr. of patients with at least one shunt dysfunction/obstruction		Nr. of patients with at least one infection-related complication		Nr. of death	
	VPS	VAS	VPS	VAS	VPS	VAS	VPS	VAS
Ignelzi and Kirsch	67 (56%)	85 (48%)	59 (52%)	74 (42%)	7 (6%)	11 (6%)	NA	NA
Olsen and Frykberg	59 (85%)	56 (54%)	60 (87%)	71 (69%)	16 (23%)	31 (30%)	8 (13%)	35 (34%)
Fernell et al.%	NA	NA	NA	NA	NA	NA	7 (5%)	8 (10%)
Keucher and Maeley%	NA	NA	NA	NA	NA	NA	9 (11%)	15 (10%)
Lam and Villemure	28 (38%)	16 (33%)	25 (34%)	8 (16%)	NA	NA	NA	NA
Borgbjerg et al	141(38%)	264 (51%)	NA	NA	NA	NA	NA	NA
McGovern et al	17 (11%)	10 (33%)	14 (9%)	2 (7%)	3 (2%)	0 (0%)	NA	NA
Hung et al	100 (29%)	16 (11%)	53 (15%)	10 (7%)	10 (3%)	2 (1%)	NA	NA
Rymarczuk et al	248 (54%)	34 (40%)	NA	NA	18 (4%)	0 (0%)	NA	NA

%, this study reported the total amount of outcome occurrence thus not allowing to extrapolate in how many patients the selected outcomes of interest occurred or did not occur. VPS indicates ventriculoperitoneal shunt; VAS, ventriculoatrial shunt.

### Outcomes

**Figure 2:**
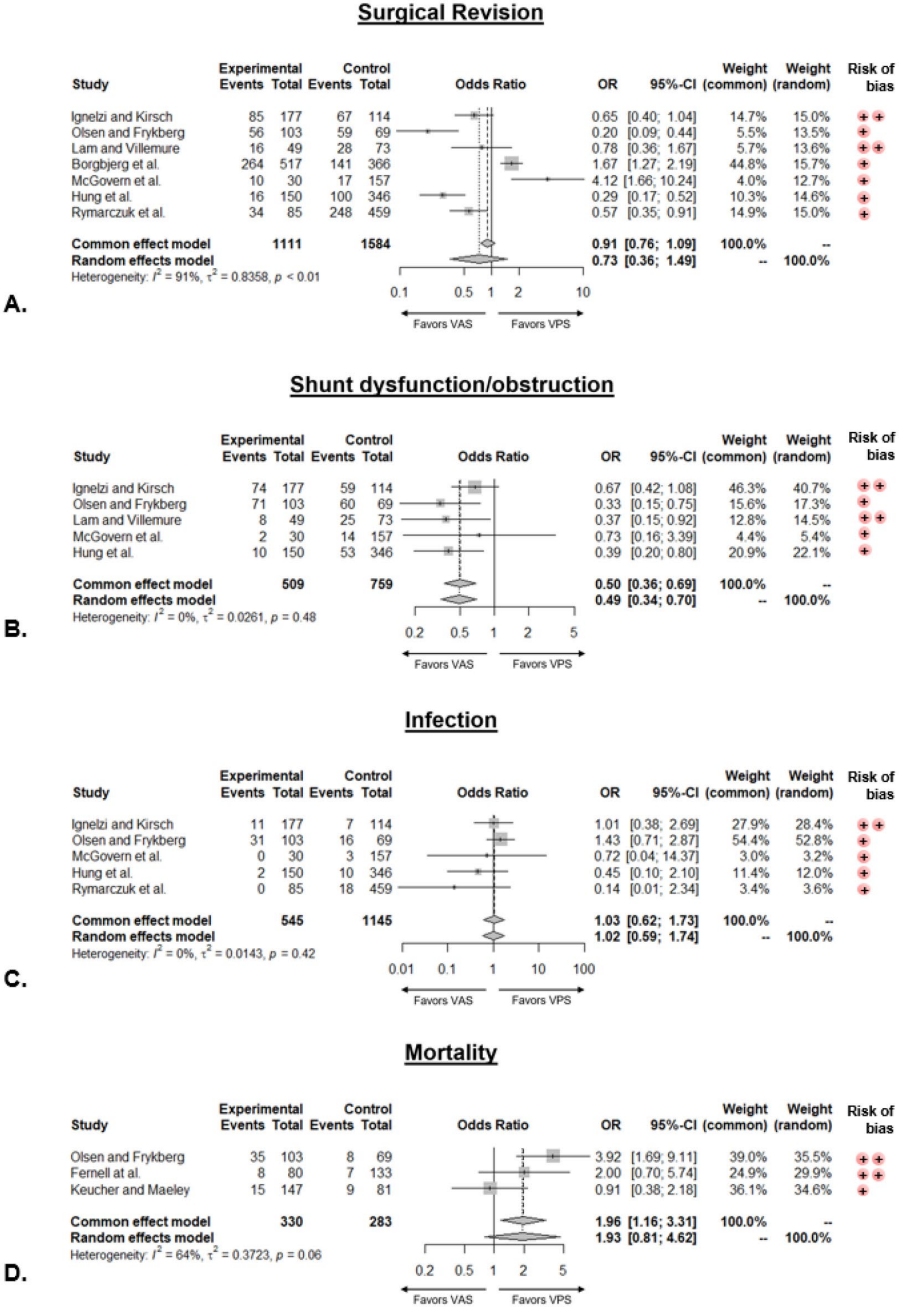
Forest plots of the pooled analysis of the investigated outcome variables. *VAS* indicates vantriculoatrial shunt, *VPS* ventriculoperitoneal shunt. (++) indicates critical risk of bias, whereas (+) serious risk of bias.

In Fig. 2 the results of the meta-analysis of the pooled study findings for the investigated outcomes are reported by means of forest plots.

Surgical revision: seven studies reported the frequency of at least one surgical revision^9–15^. 481 events were observed in the VAS group (n = 1111) and 660 in the VPS group (n = 1584). The risk for revision was not significantly different between the two groups; nonetheless, the heterogeneity between the studies was high [odds ratio (OR) 0.73, 95%-CI 0.36–1.49, I^2^ 91%].

Only one pediatric study provided data on surgical revision, indicating a significantly lower risk in the VAS group (OR 0.20, 95%-CI 0.09–0.44)^10^. Among adult studies all three reported on surgical revision. However, the risk did not significantly differ between VPS and VAS groups (OR 0.95, 95%-CI 0.21–4.26, I^2^ = 91%)^11,13,14^.

Shunt dysfunction/obstruction: five studies reported the frequency of at least one shunt dysfunction/obstruction^9–11,13,14^. 165 events were observed in the VAS group (n = 509) and 211 in the VPS group (n = 759). The risk for shunt dysfunction/obstruction was significantly lower in the VAS group (OR 0.49, 95%-CI 0.34–0.70, I^2^ 0%). Only one pediatric study provided data on shunt dysfunction/obstruction, showing a significantly lower risk in the VAS group (OR 0.33, 95%-CI 0.15–0.75)^10^. Among adult patients all three studies reported on shunt dysfunction/obstruction^11,13,14^. The risk was significantly lower in the VAS group (OR 0.42, 95%-CI 0.25–0.70, I^2^ = 0%).

Infection: five studies reported the frequency of at least one infection^9,10,13–15^. 44 events were observed in the VAS (n = 545) and 54 in the VPS group (n = 1145). The risk for infection was not significantly different between the two groups (OR 1.02, 95%-CI 0.59–1.74, I^2^ 0%).

Only one pediatric study reported infection data, showing no significant difference in risk between the VPS and VAS groups (OR 1.43, 95%-CI 0.71–2.87)^10^. Among adult patients only two studies reported infection information^13,14^. The risk did not significantly differ between the VPS and VAS groups (OR 0.50, 95%-CI 0.13–1.95, I^2^ = 0%).

Mortality: three studies reported the frequency of death^10,16,17^. 8 events were observed in the VAS group (n = 330) and 22 in the VPS group (n = 283), The risk of death was not significantly different between the two groups; nonetheless, the heterogeneity between the studies was high (OR 1.93, 95%-CI 0.81–4.62, I^2^ 64%). Three pediatric studies provided mortality data, showing no significant difference in risk between the VPS and VAS groups (OR 1.93, 95%-CI 0.81–4.62, I^2^ = 64%)^10,16,17^. No studies focusing on adult patients reported mortality information.

### Publication Bias

Funnel plots of the investigated outcomes can be found in the Supplementary Content 2 – Funnel Plots.

## Discussion

Firstly described in historical medical works by Galen and Hippocrates^18^, hydrocephalus is a common disorder of CSF physiology resulting in abnormal expansion of the cerebral ventricles, affecting an estimated number of 85 per 100,000 individuals in the general population ranging from children to the elderly^19^. The first CSF diversion system was proposed by Mikulicz in the 1893 with a permanent ventriculo-subarachnoid-subgaleal shunt, representing simultaneously a ventriculostomy and a drainage into an extrathecal low pressure compartment^18^. Since then, several attempts were made to find a practical and reliable solution for the drainage of the exceeded CSF. In 1946 the first silicone implant for human usage was introduced, but only ten years later in 1956 it was firstly utilized for the development of a CSF shunt by Holter and Pudenz^20^. Almost in the same time period, the creation of a valve system capable of adjustment of the opening-pressure and consequently preventing CSF reflux in the brain started a new era of surgical treatments for hydrocephalus, leading to the development of the VAS in the 1960s^1^ and of the VPS in the 1970s^2^.

In 1970s and 1980s, different studies by Little et al., Olsen and Frykberg, and Keucher and Mealey reported the inferiority of VAS compared to VPS in pediatric population. It was reported that VAS had a higher mortality and required more lengthening revisions because of child’s growth, although the infection and dysfunction rate was similar between the two techniques^10,21,22^. After these studies, there was a gradual switching from VAS to VPS, even though few works comparing the two techniques in the adult population were reported. In 2014 McGovernor et al. compared the safety of VAS and VPS in adult and elderly patients with idiopathic NPH, highlighting the absence of the risk of surgical revisions to lengthen the distal shunt in the adult population^13^. Additionally, the frequent use of anticoagulant or antiplatelet therapies in the elderly contributed to lower the risk of distal thrombi and/or cardiopulmonary complications. In conclusion, the authors suggested that VAS was as safe as VPS in the surgical treatment of idiopathic NPH^13^. In addition, compared to the 1970s and 1980s studies, there was a relevant improvement in infection control and intraoperative imaging enabling a safer catheter placement in the right atrium. Despite this, in recent decades, VAS has generally been considered as a last resort surgical option in both adult and pediatric population. Currently, VPS represents the first treatment option in almost all patient with hydrocephalus, even in challenging cases of history of abdominal surgery or increased abdominal pressure due to obesity^23,24^. In addition, during the last years the new generation of neurosurgeons has become more familiar and confident with VPS technique, thus explaining the underutilization of VAS. Aside from these statements, the final technical choice relies eventually on surgeon’s preference and expertise.

In this context, this study aims to evaluate the complication profile of VAS e VPS, including studies that reported comparative data between the two techniques. Through a comprehensive systematic review and meta-analysis of the available literature until September 2023, surgical revisions, shunt dysfunction/obstruction, infection, and mortality were assessed among 3197 patients, with a heterogeneous etiology and age-population of hydrocephalus (Table 1).

The literature does not provide high evidence regarding which type of shunt requires fewer revisions. Puca et al. reported revision rates of 28% for VPS and 27% for VAS^25^. According to Hung et al., the probability of shunt obstruction and shunt revision was lower in patients with VAS than in patients with VPS^14^. In this study, there were no statistical differences regarding the need of at least one surgical revision of the shunt system (OR 0.91; 95% CI 0.76–1.09). Additionally, it was observed a lower risk of shunt dysfunction/obstruction variable in the VAS group (OR 0.50; 95% CI 0.36–0.69). Furthermore, it has not been identified statistical difference on the occurrence of at least one infection-related complication (OR 1.03; 95% CI 0.62–1.73) between the VPS and VAS groups. These results confirm data reported in studies available from the literature: L.B. Oliveira et al. reported an infection rate of 5% (95% CI: 3–7%)^3^; Merkler et al. reported an infection rate of 6.1% (95% CI: 5.7–6.5%) for VPS^26^. On the other hand, there was a higher mortality in the VAS group (OR 1.96; 95% CI 1.16–3.31), even though this result comes from the analysis of three non-recent studies, from the years 1979, 1983 and 1985^10,16,17^.

A recent meta-analysis on VAS complication, including 52 studies and involving 2862 patients, showed an estimated risk of 0% for glomerulonephritis, intracranial hemorrhage, hygroma, cardiac complications, pulmonary complications, and shunt-related mortality^3^.

Performing a literature review limited to the last 10 years from PubMed/MEDLINE and Embase regarding patients treated with VAS as primary treatment, it was observed that the main reasons for this surgical choice were previous abdominal surgery, abdominal infections, and obesity. Considering that abdominal surgeries are more common in adult patients, and the very high incidence of obesity, it is appropriate to evaluate the possibility of VAS in the treatment of hydrocephalus for this patient profile.

VAS is a safe surgical option for hydrocephalus. In this study, it was observed a lower risk of shunt dysfunction/obstruction in the VAS group, and there were no statistical differences regarding the occurrence of at least one infection-related complication. This data could change with improvements of the technique, overall quality, and availability of diagnostic equipment and interventional radiologists. Our findings suggest that VAS is a safe alternative when VPS is not feasible. Nonetheless, further randomized studies are required to establish the real benefit of one type of shunt over the other.

### Limitations

Some important limitations should be considered. First, as mentioned above, in some of the included studies the follow-up time differed significantly among the two surgical groups of patients that underwent VAS and VPS treatment; in this context the variability of the outcome rates is difficult to compare. Second, the analysis included a heterogeneous population with both adults and pediatric patients. Lastly, in recent years VAS has been often considered as second treatment option due to technical preferences and biases of neurosurgeons. This is reflected in the studies included in this work, representing an uncontrollable source of confounders, and therefore limiting the comparative analysis.

## Conclusion

VAS continues to be a valuable surgical option. The results of this study suggest that VAS is a safe surgical option. Although there is a high heterogeneity between the examined studies, the risk for shunt dysfuction/obstruction is significantly lower in the VAS group and on the other hand, the risk of infection, revision and death were not significantly different between the two groups. The choice between these two techniques must be tailored to the specific characteristics of the patient. In particular, VAS may be a valuable option in cases of previous abdominal surgery, abdominal infections and obesity. The new generations of neurosurgeons are encouraged to learn both the surgical procedures in order the best option for every patient. Given the limitations outlined above, it is crucial to interpret the results with caution. Encouraging future research with randomized clinical trials is essential to overcome these limitations and improve our understanding of the indications and complications of VAS. In particular, we suggest conducting trials with similar patient profiles, analyzing the differences in the surgery time duration between the two techniques, and having follow-up data as long-term as possible.

### Supplementary Information

Supplementary Information 1.

Supplementary Information 2.

## Data Availability

Data or information needed to re-produce the findings presented are available from the corresponding author upon reasonable request.

## Abbreviations

CSF: Cerebrospinal fluid
VPS: Ventriculoperitoneal Shunt
VAS: Ventriculoatrial shunt
NPH: Normal-pressure hydrocephalus
CI: Confidence interval
OR: Odds ratio
