# Supplementary material for: Ventriculoatrial shunt remains a safe surgical alternative for hydrocephalus: a systematic review and meta-analysis
Source: Sci Rep. 2024 Aug 9;14:18460. doi: 10.1038/s41598-024-62366-8 (PMC11310213; doi:10.1038/s41598-024-62366-8)
Supplement: Supplementary file 1 — Supplementary Information 1. [file 41598_2024_62366_MOESM1_ESM.docx]

**Supplementary Content 1 - Detailed Search Strategy**

| **Database** | **Search terms** | **Exceptions** | **Strategy** | **Results** |
| --- | --- | --- | --- | --- |
| Pubmed/MEDLINE | Ventriculoatrial; shunt; ventriculo-atrial; ventricular atrial; VA shunt | - | (“ventriculoatrial” OR “ventriculo-atrial” OR “ventricular atrial” OR “VA”**AND** “shunt”) | 1902 |
| Embase | Ventriculoatrial; shunt; ventriculo-atrial; ventricular atrial; VA shunt | - | (“ventriculoatrial” OR “ventriculo-atrial” OR “ventricular atrial” OR “VA”**AND** “shunt”) | 3711 |
| Cochrane Library | Ventriculoatrial; shunt; ventriculo-atrial; ventricular atrial; VA shunt | - | (“ventriculoatrial” OR “ventriculo-atrial” OR “ventricular atrial” OR “VA”**AND** “shunt”) | 4969 |

**Supplementary Table 1.** Search strategies stratified by databases.
