# Supplementary figures and images for: Ventriculoatrial shunt remains a safe surgical alternative for hydrocephalus: a systematic review and meta-analysis
Source: Sci Rep. 2024 Aug 9;14:18460. doi: 10.1038/s41598-024-62366-8 (PMC11310213; doi:10.1038/s41598-024-62366-8)

**Supplementary Content 2** – Funnel Plots


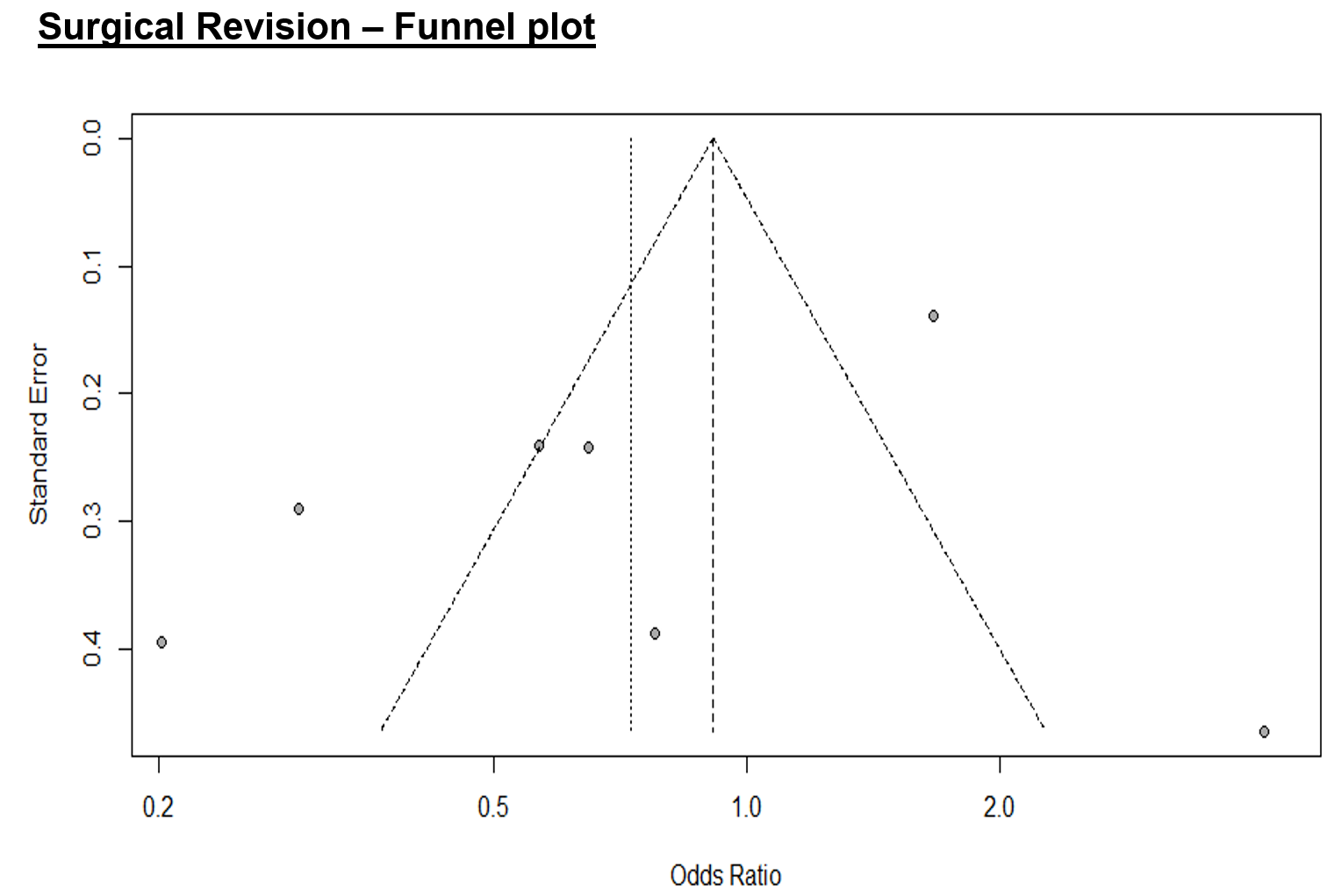


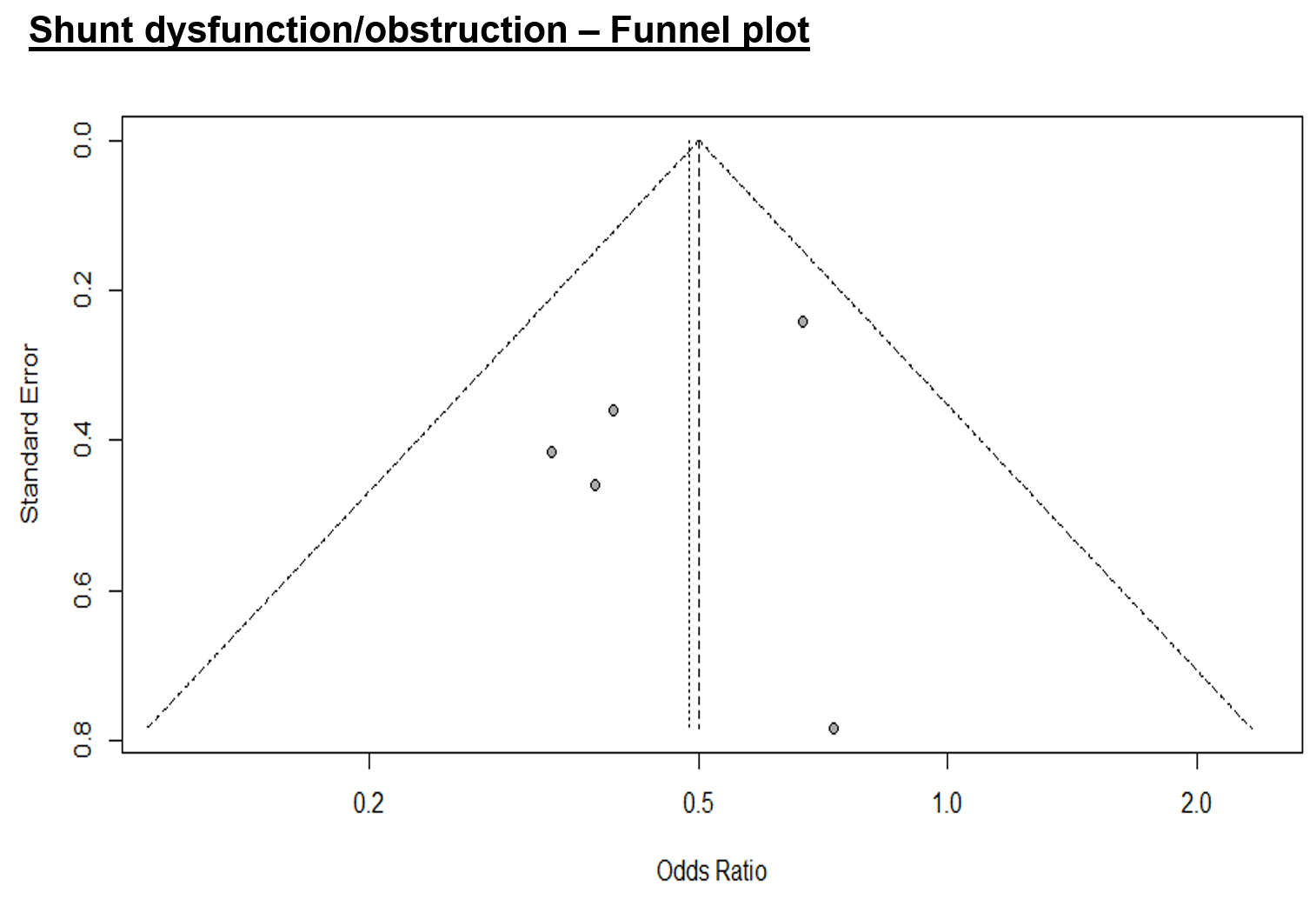


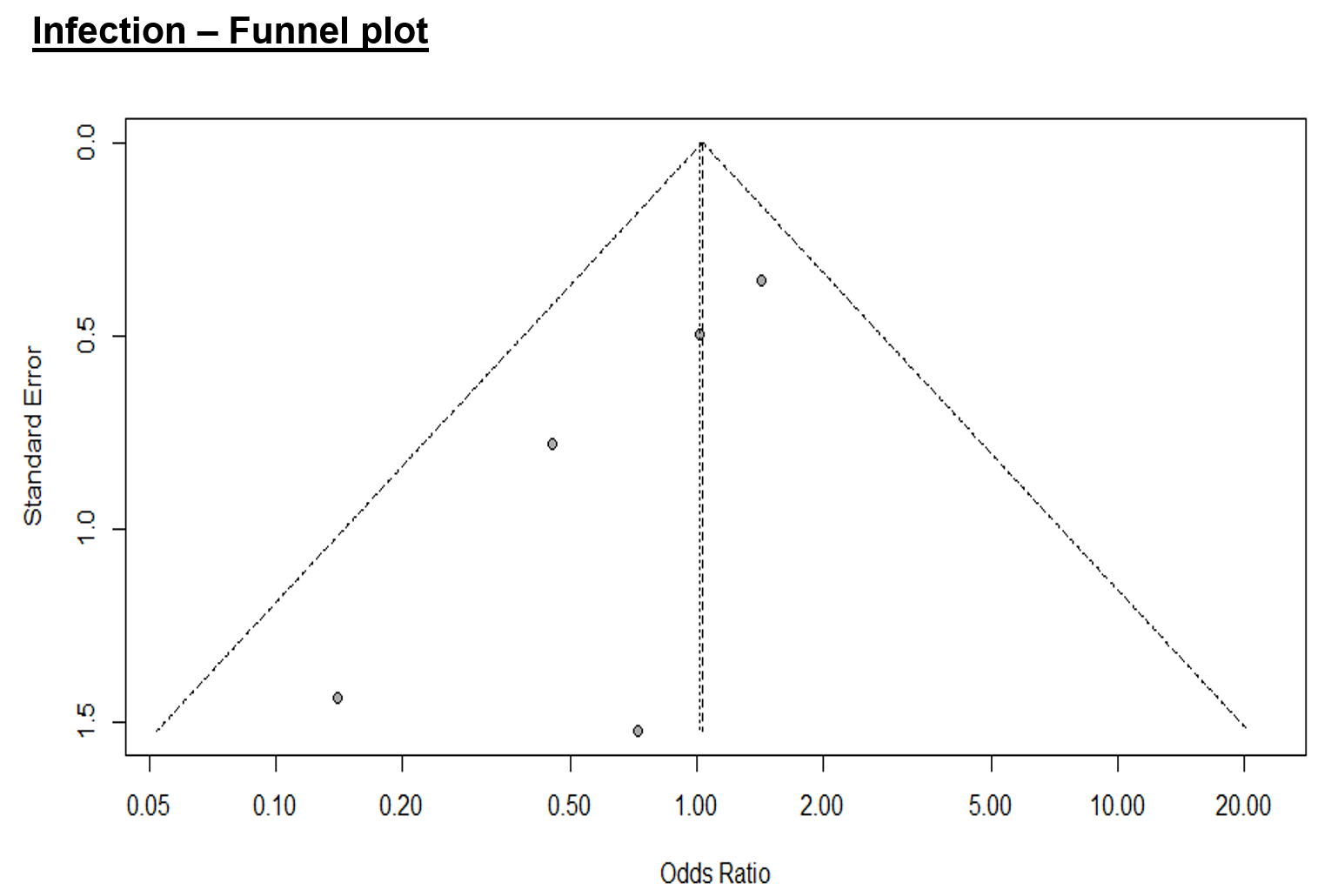


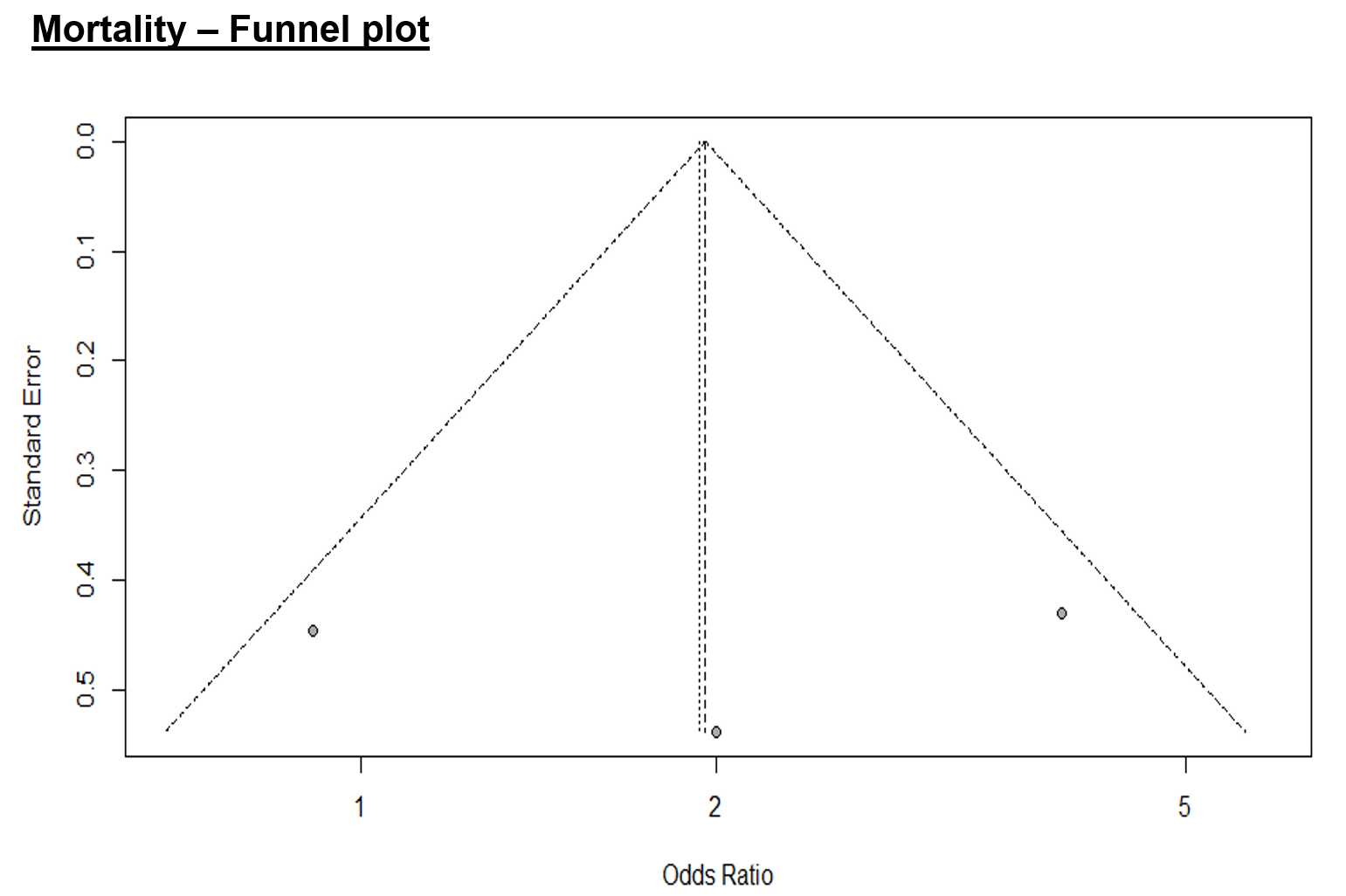

Supplement: Supplementary file 2 — Supplementary Information 2. [file 41598_2024_62366_MOESM2_ESM.docx]
